# Nuclear Factor-κB Signaling in Murine Models of Malignant Pleural Mesothelioma

**DOI:** 10.3390/cancers18182990

**Published:** 2026-09-16

**Authors:** Benteng Deng, Ioanna Giopanou, Lori Asarian, Ioannis Lilis, Jianlong Jia, Marina Lianou, Dimitris Simoglou, Giannoula Ntaliarda, Tonia A. Adamide, Isis E. Fernandez, Georgios T. Stathopoulos

**Affiliations:** 1 Institute of Lung Health and Immunity (LHI), Comprehensive Pneumology Center (CPC), Helmholtz Zentrum München, German Center for Lung Research (DZL), 81377 Munich, Bavaria, Germany; benteng.deng@helmholtz-munich.de (B.D.);; 2 Department of Medicine V, LMU Klinikum, Ludwig-Maximilians-Universität München, 81377 Munich, Bavaria, Germany; 3 Laboratory for Molecular Respiratory Carcinogenesis, Department of Physiology, Faculty of Medicine, University of Patras, 26504 Rio, Achaia, Greece; 4 Department of Pulmonary Medicine, Nicosia General Hospital, Strovolos 2031, Nicosia, Cyprus; 5 Medical School, University of Cyprus, Aglantzia 2029, Nicosia, Cyprus

**Keywords:** malignant pleural mesothelioma, NF-κB, IKKα, IKKβ, Chuk, Ikbkb, shRNA, RNA-seq, murine tumor models

## Abstract

Background: Malignant pleural mesothelioma (MPM) is an aggressive asbestos-associated cancer in which inflammatory pathways may contribute to tumor behavior. NF-κB signaling is a central mediator of inflammation and cell survival, but the relative contributions of its associated enzymes, IKKα- and IKKβ, in MPM models remain incompletely understood. Methods: We characterized the involvement of IKKα- and IKKβ in tumor formation using several in vitro approaches and the intrapleural or subcutaneous delivery of three murine MPM cell models (AB2, AE17 and KPM1). The experimental approaches included shRNA silencing of *Chuk* and *Ikbkb*—the genes encoding the activity of IKKα- and IKKβ—and tumor growth, apoptosis and colony formation measurements. Exploratory pathway and transcriptional analyses were carried out following RNA-sequencing of our murine cell lines and a publicly available human gene expression dataset was also analyzed to provide complementary evidence. Results: Activation of the IKKα- and IKKβ was confirmed in the AB2, AE17 and KPM1 cell lines. Silencing both *Chuk* and *Ikbkb* decreased target and cross-target transcripts and metabolic growth. Importantly, *Chuk* silencing was associated with increased early apoptotic staining in AE17 and KPM1 cells, increased late apoptotic staining in AE17 cells and reduced colony formation in all cell models. Furthermore, *Chuk*-associated effects on tumor burden and activity were more notable than *Ikbkb*-associated effects in both intrapleural and subcutaneous tumor formation parameters. Finally, while canonical NF-κB gene-set clustering in both human and murine tumor samples identified specific and shared pathways and transcriptional profiles supporting the in vitro murine assay findings, the analyses remained exploratory and did not specifically establish target dependency. Conclusions: Our data support the involvement of IKK/NF-κB-associated signaling in cell survival, clonogenicity and tumor growth in several MPM murine cell models and identify *Chuk*-related signaling as a promising target in future studies.

## Simple Summary

Malignant pleural mesothelioma (MPM) is an aggressive cancer linked to asbestos exposure. To shed light on the underlying mechanisms, we examined the roles of NF-κB-regulatory enzymes IKKα and IKKβ in three murine mesothelioma cell models. To this end, we targeted the activity of *Chuk* and *Ikbkb*, the two genes that encode the activity of IKKα and IKKβ, respectively, using intrapleural and subcutaneous murine tumor models and a battery of in vitro functional assays. While decreasing the activity of both genes decreased growth-associated tumor metabolic activity, *Chuk* silencing led to more apoptosis, impaired colony formation and decreased tumor formation. Exploratory analyses of a publicly available human mesothelioma gene-expression dataset and our murine RNA-sequencing data complemented the in vitro data at the pathway and transcriptional levels. These findings confirm the importance of IKK/NF-κB signaling complex in MPM and identify *Chuk* as a promising therapeutic target.

## 1. Introduction

Malignant pleural mesothelioma (MPM), an uncommon but aggressive cancer that develops in the visceral or parietal lung pleura, is known to be associated with exposures to asbestos and related mineral fibers [1]. Its long latency and chronic inflammatory pathogenesis contribute to a substantial clinical burden [2]. In GLOBOCAN 2022 data, Fu and colleagues estimated 30,633 new mesothelioma cases and 25,371 deaths worldwide in 2022, with Europe accounting for nearly half of incident cases and marked regional and sex disparities [3]. Although this burden is much smaller than the almost 2.5 million lung cancer cases estimated globally in 2022 [4], MPM remains a major public health problem because of its poor prognosis and limited treatment options. Current management remains challenging despite platinum-pemetrexed chemotherapy and guideline-supported first-line immune-checkpoint blockade for selected patients with unresectable pleural mesothelioma [5,6,7]. Median survival has historically been reported at approximately 8–14 months from diagnosis, although outcomes vary by treatment setting and histological subtype [8]. Sarcomatoid or non-epithelioid tumors generally have less favorable outcomes than epithelioid tumors [9,10]. Molecular heterogeneity may also influence outcome and treatment response; genomic, transcriptomic, multi-omic, and pharmacogenomic studies therefore provide a rationale for biologically informed patient stratification and treatment development [11,12,13,14,15].

Nuclear factor-κB (NF-κB), a transcription factor implicated in tumors in various tissues [16,17,18,19], is activated by several inflammatory stimuli, such as cytokines, bacteria, viruses, radiation and mutated oncogenes. Its activation leads to genomic stress and alterations in cell-survival, immune and proliferative functions [20] that can trigger signaling via canonical and non-canonical pathways. Whereas canonical pathway triggers, such as infection and inflammatory signals, result in rapid activation of NF-κB and its downstream target genes, non-canonical pathway signaling occurs over a longer timeframe and involves de novo protein synthesis and dimer formation. The identification of the IκB inhibitory proteins and IκB kinase (IKK) complexes provided important insights into the mechanistic and biological differences between the canonical and non-canonical pathways. Specifically, canonical signaling leads to the phosphorylation and activation of the NF-κB essential modulator (NEMO) and IKKα:IKKβ complex, and the subsequent release and nuclear translocation of RelA/p50 dimers [20]. On the other hand, non-canonical signaling initiated by upstream signaling converges on NF-κB-inducing kinase (NIK)-dependent IKKα activity, independent of NEMO and IKKβ, and leads to p100 phosphorylation and subsequent nuclear signaling via RelB/p52 dimers [21,22,23].

Despite the 70% structural homology in their structural domains, important differences between IKKα and IKKβ that may influence their role in health and disease have been identified [24,25]. While both are part of the canonical pathway and may be activated by innate and adaptive immune responses or exogenous mediators, such as TNF-α and LPS, IKKβ’s kinase activity is 20–50-fold higher than that of IKKα [25,26], suggesting a more prominent role in the emergency, rapid immune responses. In addition to a role within the canonical signaling, however, IKKα can influence immune cellular processes not only via non-canonical NF-κB pathways, but also via NF-κB-independent pathways [23], giving it a broader arsenal in modulating cellular immunity. Finally, because mutations in genes encoding both IKKα and IKKβ (*Chuk* and *Ikbkb*, respectively) have been associated with several malignancies and are involved in alterations in tumor microenvironment [27,28], here we examined both as part of our approach to identify the mechanisms underlying MPM.

Several inflammatory mediators that are potent activators of NF-κB signaling pathways described above are released in response to asbestos-associated mesothelial injury and have been linked experimentally to the NF-κB-dependent survival of damaged mesothelial cells [29]. Although previous studies have examined these inflammatory pathways and circulating biomarkers (e.g., RANTES, mesothelin-family proteins, ALDH1A3-associated STAT3/NF-κB signaling, SOCS-1 signaling) in the context of MPM and how they may be affected by chemotherapy [30,31], the specific contributions of IKKα- and IKKβ-linked signaling to MPM remain incompletely understood. To this end, we evaluated the phenotypic consequences associated with genetic manipulations of *Chuk* and *Ikbkb* in chemically induced or genetically engineered murine MPM models. We focused on IKKα and IKKβ expression and localization, apoptosis and clonogenic assays and RNA-seq profiling in murine cell lines and complemented these findings with exploratory pathway analyses of a publicly available human dataset.

## 2. Materials and Methods

### 2.1. Mice

C57BL/6 (#000664) and BALB/c (#001026) mice were obtained from Jackson Laboratories (Bar Harbor, ME, USA). B6.129S4-*Kras*^tm4Tyj/J mice (hereafter *Kras*^G12D; #008179) [32] and B6.129P2-*Trp53*^tm1Brn/J mice (hereafter *Trp53*^f/f; #008462) [33] were also obtained from Jackson Laboratories, but subsequently bred on the C57BL/6 background at the University of Patras Center for Animal Models of Disease. Experiments were approved by the Prefecture of Western Greece’s Veterinary Administration (approval 118018/578; 30 April 2014) and conducted according to Directive 2010/63/EU. Mice were matched for sex, weight (20–25 g), and age (6–12 weeks) prior to experimental manipulations.

### 2.2. Cell Lines, Culture Conditions, and shRNA Constructs

BALB/c mouse AB2 and C57BL/6 mouse AE17 MPM cells were a gift from Dr. YC Gary Lee (University of Western Australia, Perth, Australia) [34]. C57BL/6 mouse KPM1 cells, primary WT pleural mesothelial cells (PMCs) and urethane-induced lung adenocarcinoma (CULA) *Trp53*^f/f^ allele cells were generated in our laboratory, using previously described protocols for the KPM1 line [35], for PMC [35] and by using a Cre-recombinase conditional approach [36], respectively. Public repository identifiers for the cell lines were as follows: CVCL_LJ79, CVCL_4408 and CVCL_A9KU, for AB2, AE17 and CULA, respectively. The KPM1 line is deposited at the Laboratory for Molecular Respiratory Carcinogenesis cell-line facility (Patras, Greece) and is available upon request. Because they have been previously characterized [17,35,37], PMC and CULA cells were only used as controls for the verification of the *Kras* or *Trp53* mutation status of the AE17, AB2 and KPM1 cell lines. The human cell lines MET5A, MSTO-211H, and ZL34 (public repository identifiers: CVCL_3749, CVCL_1430, and CVCL_5906, respectively) were only used as controls for NF-κB-subunit expression analysis. All cell lines tested negative for mycoplasma.

Human cell lines were cultured at 37 °C in 5% CO_2_ in DMEM (MET5A and ZL34) or RPMI 1640 (MSTO-211H) and supplemented with 10% fetal bovine serum, 2 mM L-glutamine, 1 mM pyruvate, 100 U/mL penicillin, and 100 μg/mL streptomycin. Murine pleural mesothelial cells (AE17, AB2 and KPM1) were cultured in DMEM as described above for the human cell lines and then used following shRNA transfection for the further characterization of MPM tumor cell models. For stable lentiviral shRNA transfection, 10^5^ tumor cells were treated with lentiviral shRNA particles according to the manufacturer’s protocol (Santa Cruz Biotechnology, Dallas, TX, USA). For *Chuk* and *Ikbkb*, a pool of three target-specific sequences was used, whereas a proprietary, scrambled sequence (shC) was used as a control (Supplementary Table S1). GFP was incorporated in the vectors and was induced upon successful transduction. Non-transfected cells were then selected using puromycin (2–10 μg/mL; Gibco, Invitrogen, Carlsbad, CA, USA). For the characterization of tumor models, cells were harvested, stained with trypan blue and counted using a hemocytometer. Cell suspensions with >95% viability were then used for in vitro studies or administered to mice, as described previously [35].

### 2.3. Metabolic Activity, Colony Formation, and Apoptosis Assays

Growth-associated metabolic activity of the various cell lines was assessed using the 3-(4,5-dimethylthiazol-2-yl)-2,5-diphenyltetrazolium bromide (MTT) reduction assay. Following shRNA transfection, cell lines (AB2, AE17 and KPM1) were seeded (3 × 10^3^ cells per well in 96-well plates, n = 6 per shRNA transcript) and cultured for four days. Each day, 10 μL of MTT (Sigma, St. Louis, MO, USA) working solution (5 mM MTT in PBS) was added to each well. Four hours later, 100 μL acidified isopropanol was added, followed by sediment solubilization. Absorbance (492 nm) was measured in a MR-96A microplate reader (Mindray, Shenzhen, China). Absorbances of wells with DMEM alone, in the absence of cells, were used as background and were subtracted from the readings of experimental well. Corrected absorbances were subsequently used for statistical analyses.

For colony formation assays, cells were seeded in 6-well plates (100 cells/well for AB2 and AE17 cell lines, n = 6 and 4, respectively; 300 cell/well for KPM1, n = 3) were closely monitored for 2 weeks until colony formation. Colonies were then fixed and stained with 0.5 g Crystal Violet (0.05% *w*/*v*) (TargetMol, Boston, MA, USA) for 20 min at room temperature and counted.

Detection of early and late apoptosis was done using an annexin-V-7AAD assay. Briefly, 10^6^ cells in 100 μL Annexin-V 1x buffer were incubated at room temperature with Annexin-V (ImmunoTools, Friesoythe, Germany; 31490014, 1:200 dilution) and 7AAD (eBiosience Inc., San Diego, CA, USA; E00031-1632, 1:20 dilution), for 15 min. Late apoptotic cells were characterized as double positive Annexin-V/7AAD, while early apoptotic cells as represented by Annexin V+/7-AAD- labels. Statistical analyses were done on a set of three replicates per cell model. Cell counts were carried out using CyFlow ML (Partec, Darmstadt, Germany) and analyses with FlowJo software v10.10.1 (FlowJo, Ashland, OR, USA).

### 2.4. PCR and RNA Sequencing

Total RNA was isolated using TRIzol reagent (Invitrogen, Carlsbad, CA, USA) and the RNeasy kit (Qiagen, Hilden, Germany) and reverse transcribed using SuperScript III (Invitrogen, Carlsbad, CA, USA). To identify the *Trp53* mutation status in PMC, AE17, AB2, KPM1 and CULA cells, RT-PCR was performed using SYBR Green Master Mix on a StepOnePlus instrument (Applied Biosystems, Carlsbad, CA, USA). Following shRNA transfection, the extent of gene silencing was reported following quantitative RT-PCR (qPCR). The absence of mycoplasma in the cell culture experiments was ensured with a standard PCR reaction. All primers are listed in Supplementary Table S3. Primers were used according to previously published protocols to ensure maximum efficiency [38,39]. For the qPCR, relative expression was normalized to *Gusb* and calculated against the indicated control condition using the 2^−ΔCt^ method [40].

For RNA-seq, total RNA from shRNA-targeted AB2, AE17 and KPM1 cell lines was extracted from 106 cultured cells. Libraries were prepared from 2 μg of total RNA and single-end sequencing was carried out on Illumina Hiseq 4000 (San Diego, CA, USA). FASTQ files were aligned to GRCm38/mm10 using STAR v2.7, and read counts were generated with featureCounts/Subread before downstream differential-expression analysis in R v4.4.2 using limma v4.22.0 and edgeR v4.4.0 [41,42]. Genes with a nominal *p* < 0.05 and an absolute log2 fold change of ≥1.7 were retained for the exploratory summaries. Differentially expressed genes (DEGs) in the 3 cell lines were then analyzed for covariance among the different shRNA targets (control, *Chuk*, *Ikbkb*) and column z scores were used for hierarchical clustering. Heatmaps and PCA plots were generated in R using gplots (v3.2.0)/heatmap.2 and factoextra (v2.0.0)/fviz, respectively. Raw BAM files and processed data are deposited in the NCBI Gene Expression Omnibus under accession GSE129984 [43].

### 2.5. Intrapleural and Subcutaneous Tumor Models

For intrapleural tumor experiments, 2 × 10^5^ AE17 or KPM1 cells transfected with shRNA (control, *Chuk* or *Ikbkb*) were delivered in 100 μL PBS to isoflurane-anesthetized C57BL/6 mice. Similarly treated AB2 cells (1.5 × 10^5^) were administered to BALB/c mice. The number of mice used for each cell line and shRNA construct is listed in Supplementary Table S4. Following the administration of cell suspensions, mice were monitored during post-surgical recovery, and daily thereafter, and were sacrificed when moribund. Survival time was recorded and pleural fluid and tumors were collected. The isolated malignant pleural effusion (MPE) fluid was diluted ten-fold in red blood cells lysis buffer (155 mM NH4Cl, 12 mM NaHCO3, 0.1 mM EDTA). Total nucleated pleural cells were counted using a hemocytometer and cytospin preparations were then generated using 5 × 10^4^ cells per slide for further cytological analysis. Lungs, visceral pleural tumors, parietal pleural tumors, and chest walls were fixed in 4% paraformaldehyde overnight, and tumor burden was subsequently assessed using tumor weight.

For subcutaneous solid tumor experiments, 10^6^ cells transfected as described above were injected in the rear flanks of either C57BL/6 (KPM1 or AE17 cells) or BALB/c mice (AB2 cells). The number of mice used for each cell line and shRNA construct is listed in Supplementary Table S4. Once a week for 4 weeks, three orthogonal tumor dimensions (δ1, δ2, δ3) were performed per mouse, and tumor volumes were calculated as π*δ1*δ2*δ3/6 [44,45].

### 2.6. Histology, Immunohistochemistry, Immunofluorescence, and Immunoblotting

Whole lungs were fixed overnight in 4% paraformaldehyde and then embedded in paraffin. All primary and secondary antibodies and detection methods are listed in Supplementary Table S2.

For immunohistochemistry, 4-μm paraffin sections were deparaffinized, rehydrated, subjected to heat-induced epitope retrieval in 10 mM sodium citrate (pH 6.0), and blocked with 3% H_2_O_2_ and then 3% bovine serum albumin for endogenous peroxidase quenching and against nonspecific antibody binding, respectively. Anti-IKKα (sc-7606, rabbit polyclonal IgG (1:100, Santa Cruz Biotechnology, Santa Cruz, CA, USA) and anti-IKKβ (MA5-16154, mouse monoclonal IgG, 1:100, Invitrogen, Carlsbad, CA, USA) primary antibodies were used in an overnight incubation at 4 °C. Horseradish peroxidase (HRP) was used as the detection method, according to the manufacturer’s instructions (EnVision; Dako, Glostrup, Denmark). Sections were counterstained with Harris’s hematoxylin, dehydrated, mounted and imaged using an Axio Lab.A1 microscope with an AxioCam ERc 5s camera (Zeiss, Jena, Germany).

For immunofluorescence, cells were fixed overnight in 4% paraformaldehyde at 4 °C and then incubated with primary and secondary antibodies (Invitrogen, Carlsbad, CA, USA). Cells were counterstained with Hoechst 33258 (Sigma-Aldrich, St. Louis, MO, USA) and mounted with Mowiol 4-88 (Calbiochem, Gibbstown, NJ, USA). Primary antibody was omitted for the negative control. Imaging was carried out using an AxioObserver.D1 inverted microscope with an AxioCam ERc 5s camera (Zeiss, Jena, Germany), and image processing was done using Fiji v1.54p [46].

Nuclear and cytoplasmic extracts were prepared using the NE-PER Extraction Kit (Thermo Fisher Scientific, Waltham, MA, USA), separated on 12% SDS polyacrylamide gels using electrophoresis (PAGE), transferred to polyvinylidene difluoride membranes (PVDF, Merck Millipore, Darmstadt, Germany), probed with specific antibodies and then visualized after incubation with enhanced chemiluminescence substrate (Merck Millipore, Darmstadt, Germany).

### 2.7. Public Human Expression Data and Pathway Gene Sets

Human gene expression analysis (Affymetrix Human Genome U133 Plus 2.0 Array platform) was carried out on a publicly available dataset (GEO accession GSE51024), which included 55 MPM tumor samples and 41 paired non-tumor lung parenchyma samples [47]. Pathway Interaction Database (PID) [48] analyses used canonical and atypical NF-κB pathway gene lists. The atypical NF-κB gene list specifically identified with *Ikbkb* signaling-related genes. Unsupervised clustering of individual patients was performed using a hierarchical algorithm in the R package cluster v.2.1.6 and the expression level of the genes in the canonical or atypical lists.

### 2.8. Statistics

Unless otherwise stated, plotted data are mean ± SD or mean ± 95% confidence interval, as specified in the corresponding figure legend. All data were normally distributed and were analyzed with one- or two-way ANOVA followed by Bonferroni post hoc tests, as appropriate. Kaplan–Meier survival estimates were performed with log-rank tests. Data acquisition was carried out on blinded on samples. *p*-values were two-tailed. *p* < 0.05 was considered significant. Statistical analyses and graphs were done on Prism v5.0 (GraphPad, La Jolla, CA, USA).

## 3. Results

### 3.1. Characterization of MPM Models and NF-κB-Associated Expression Patterns

**Figure 1:**
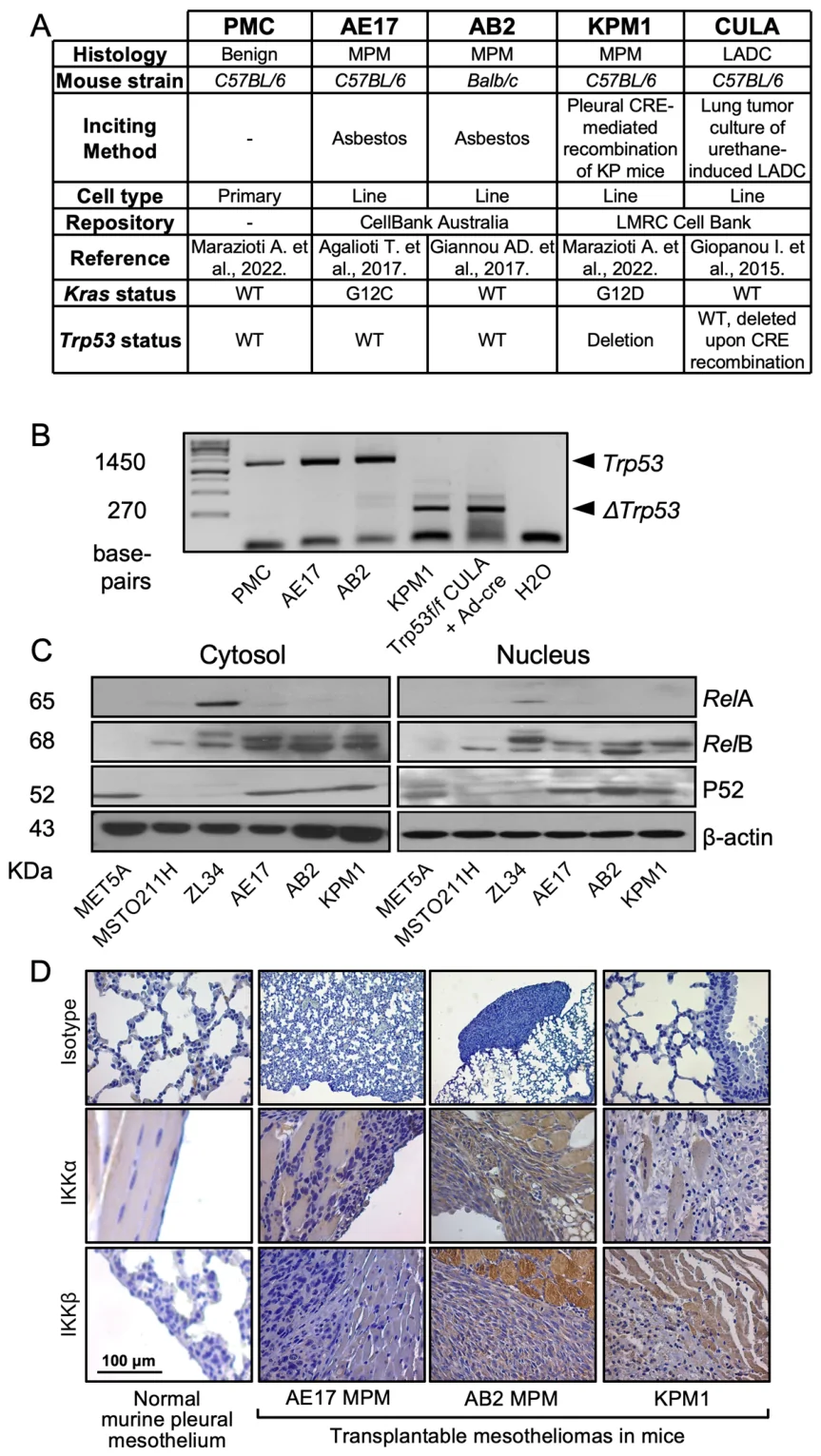
Characterization of MPM models and NF-κB-associated protein patterns. Summary of histology, origin, induction method, source/repository, reference, and *Kras*/*Trp53* status for primary mesothelial cells (PMCs), AE17, AB2, KPM1, and CULA cell lines used in the current experiments (**A**) [17,35,37,45]. Quantitative PCR showed the *Trp53^f/f^* deletion-associated 270 bp band was present in KPM1, but not AE17 and AB2 cell models. Ad-Cre-treated *Trp53^f/f^* CULA cells were used as a positive control (Supplementary Materials Figure S08) (**B**) [36]. Western blot analyses of RelA, RelB, and p52 in cytoplasmic and nuclear extracts from human MET5A, MSTO-211H, and ZL34 cells and murine AE17, AB2, and KPM1 cells. β-actin is shown as a loading control (Supplementary Materials Figures S01–S07) (**C**). Representative IKKα and IKKβ immunohistochemistry images from normal murine pleural mesothelium and tumors derived from intrapleural AE17-, AB2-, or KPM1-administration (**D**). The negative-control condition omitted primary antibody and is therefore described as a non-primary control. Scale bar, 100 μm.

Because our previous findings highlighted the importance of *Kras* and *Trp53* deletions in mice fostering malignant pleural mesothelioma [35], and because the *Trp53* mutation status is unknown in the AE17, AB2 and KPM1 cell models, we first described the origin and genotypes of these cell lines, as they will be used throughout this study (Figure 1A). Primary mesothelial cells (PMCs) and urethane-induced lung adenocarcinoma (CULA) cells were used as positive controls for presence of *Kras* and *Trp53* in our RT-PCR assays. Our results show that AB2 and AE17 cells were WT for *Trp53* mutations, whereas KPM1 cells showed a specific deletion (Figure 1B). We next examined the NF-κB-associated expression and localization patterns in both our murine cell lines (AB2, AE17 and KPM1) as well as three human MPM cell lines (MSTO211H, ZL34 and MET5A). We found that the p52 subunit was detected in both the cytoplasmic and nuclear fractions of the non-malignant MET5A cells, whereas MSTO211H cells expressed RelB in both cell compartments and ZL34 cells expressed both RelA and RelB. In the ZL34 cells, RelB appeared to be expressed higher in the nucleus and RelA overexpressed in cytoplasm (Figure 1C). The three murine cell lines AB2, AE17 and KPM1 had a similar pattern of highly expressing RelB and p52 in both the cytoplasm and the nucleus. This is consistent with a possible non-canonical NF-κB-associated pattern in both human and murine cell models. These results led us to then investigate the expression pattern for the upstream NF-κB-activating kinases (IKKα and IKKβ) in MPM. Using immunohistochemistry and samples from BALB/c and C57BL/6 mice that developed MPM upon intrapleural injection of AB2, AE17 or KPM1 cells, we identified distinct, upregulated immunoreactivity for IKKα and IKKβ (Figure 1D). In particular, in MPM developed from AE17 intrapleural cell delivery in mice, IKKα had slightly higher expression in the cytoplasm, whereas in MPM developed by AB2 intrapleural cells delivery in mice, both IKKα and IKKβ showed increased immunoreactivity at the tumor site. Finally, after KPM1 intrapleural delivery, both subunits were overexpressed, but IKKβ expression was slightly higher. Together with the subunit localization results from the Western blot studies, these findings suggest that nuclear RelB and p52 localization and possible engagement of NF-κB-associated signaling may characterize these models.

### 3.2. Effects of IKKα and IKKβ Gene Silencing on Apoptosis of MPM Cells

**Figure 2:**
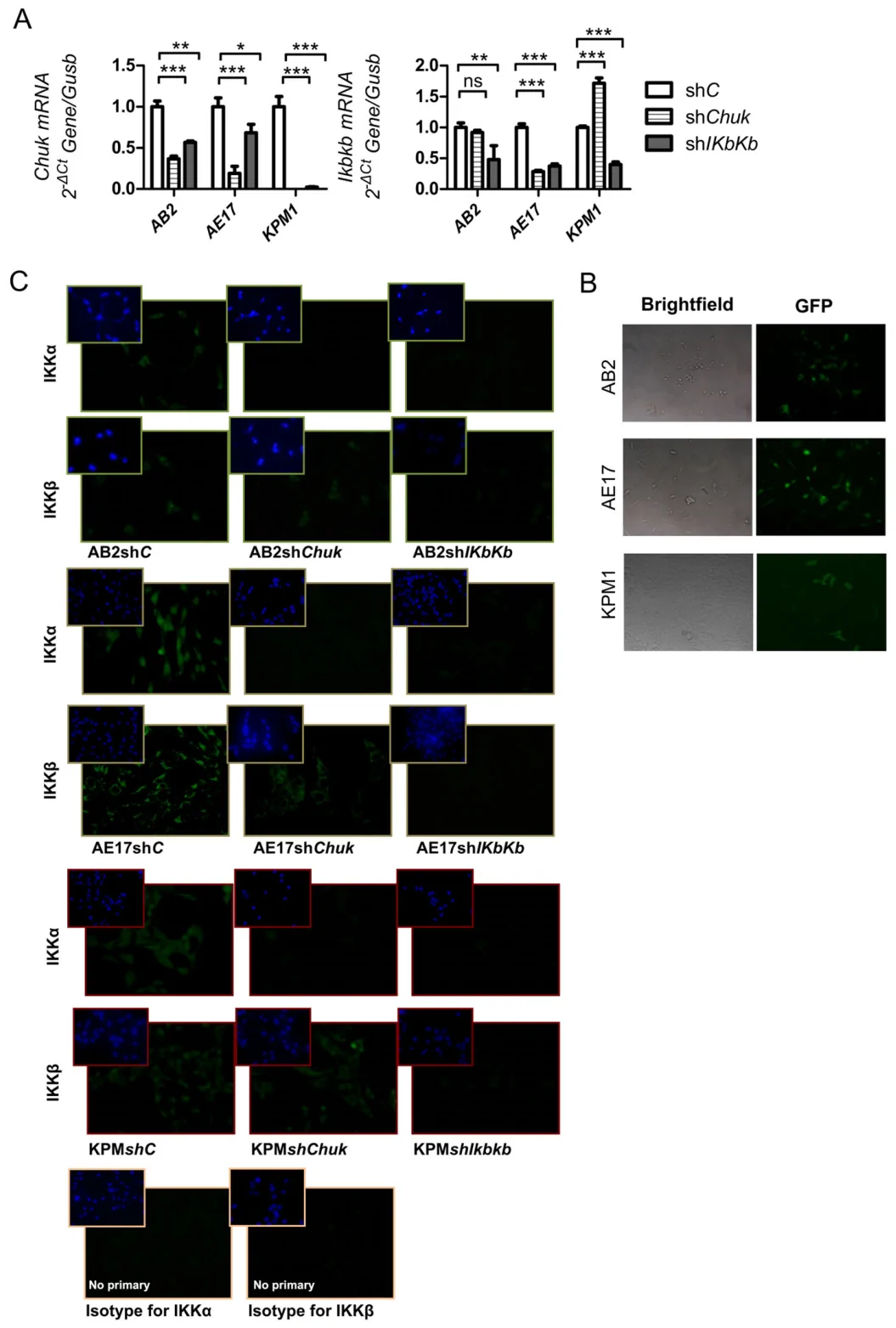
Validation of lentiviral shRNA silencing of *Chuk* and *Ikbkb* in murine MPM cell lines. Representative qPCR analysis showing expression of *Chuk* (encoding IKKα) and *Ikbkb* (encoding IKKβ) relative to *Gusb* mRNA (**A**). Representative GFP images validating vector expression/transfection. The GFP expression was the result of a built-in construct in each of the lentiviral probes. Brightfield pictures of the cell cultures (left) are provided for reference (**B**). Representative IKKα and IKKβ immunofluorescence images (green) with Hoechst 33258 nuclear counterstain (blue) following lentiviral transfection. Negative-control (bottom two panels) conditions omitted primary antibody. Lack of green fluorescence denotes absence of IKKα and IKKβ protein (**C**). Data in (**A**) are mean ± 95% confidence intervals. Significances (*** *p* < 0.001, ** *p* < 0.01, * *p* < 0.05 and ns *p* > 0.05) were obtained after two-way ANOVA with Bonferroni post hoc tests.

**Figure 3:**
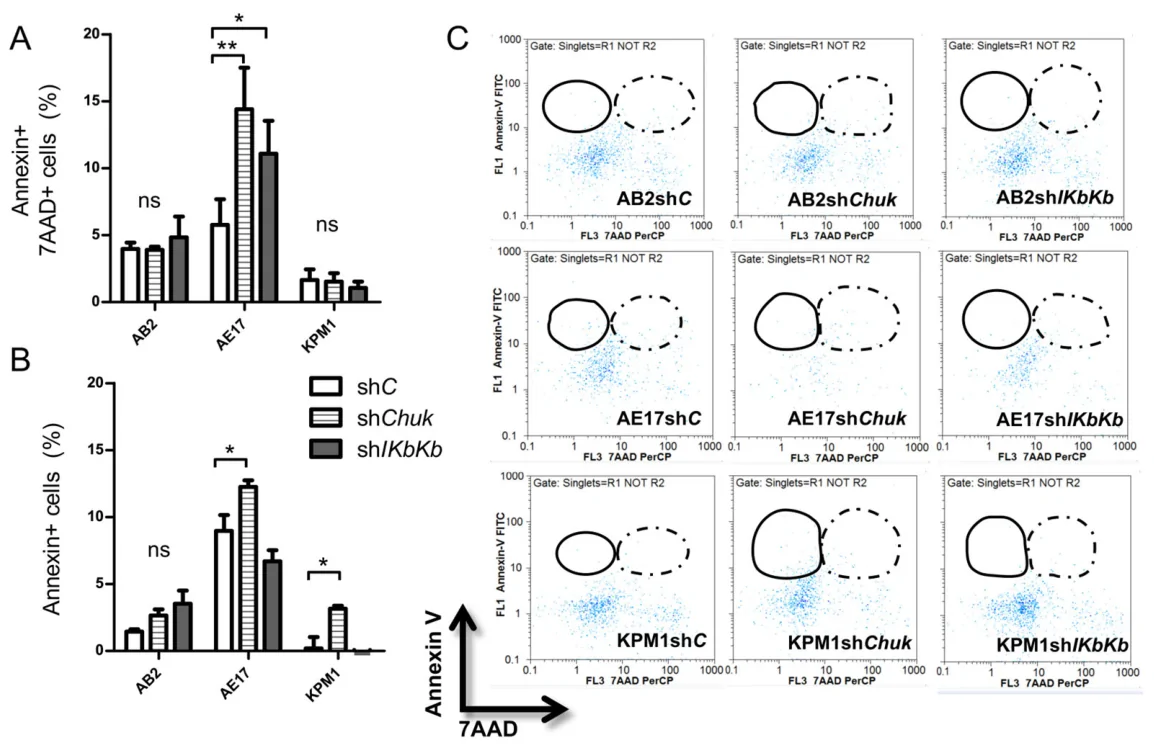
Effects of *Chuk* and *Ikbkb* silencing on apoptosis of MPM murine cell models. Apoptosis in *Chuk*- and *Ikbkb*-silenced cells was assessed by flow cytometric determination of annexin V and 7AAD staining. Annexin and 7AAD double positive cells denote late apoptosis (**A**), whereas AnnexinV+/7AAD- cells denote early apoptosis (**B**). Representative dot-plots and gating strategy from the experiments described in (**A**,**B**) are shown in (**C**), with solid and dashed circles denoting early- and late-apoptotic cells, respectively. Data are mean ± SD. Significances (** *p* < 0.01, * *p* < 0.05 and ns *p* > 0.05) were obtained after two-way ANOVA with Bonferroni post hoc tests.

To test the impact of IKK expression in tumor cell proliferation and survival we used gene silencing of *Chuk* and *Ikbkb*, the genes encoding IKKα and IKKβ expression, respectively, using lentiviral shRNAs in all three murine cell lines (AB2, AE17 and KPM1). The shRNA constructs are described in Supplementary Table S1. The extent of gene silencing in each cell line was validated with quantitative PCR (qPCR) using specific primers (Figure 2A; Supplementary Table S3) that detected *Chuk* and *Ikbkb* mRNA. GFP expression was present in all cell lines, thus validating the vector transfection (Figure 2B). We further validated IKKα and IKKβ expression following transfection using immunofluorescence (Figure 2C). Consistent with our qPCR results, we observed some cross-silencing of *Chuk* when *Ikbkb* shRNA was used, especially in the AE17 and KPM1 cell lines. It is not clear whether this was due to particular cell line characteristics or whether it was a compensatory response, given the similarities between IKKα and IKKβ. We next investigated how the decrease in *Chuk* and *Ikbkb* gene expression impacted the survival of the malignant cells using an early and late apoptotic cell death assay (Figure 3). There was no effect of *Ikbkb* gene silencing on cell survival or of *Chuk* gene silencing in AB2 cells. In contrast, *Chuk* gene silencing revealed statistically significant increases in early apoptosis in AE17 and KPM1 cell lines (Figure 3B), as well as increased late apoptosis in AE17 cells (Figure 3A). Whether these effects of *Chuk* silencing on apoptosis differed due to differences in *Kras* or *Trp53* mutation between the cell lines or because of the slight extent of cross-silencing between *Chuk* and *Ikbkb* remains an open question.

### 3.3. Effects of Chuk and Ikbkb Silencing on Tumor Cell Proliferation and Clonogenicity

**Figure 4:**
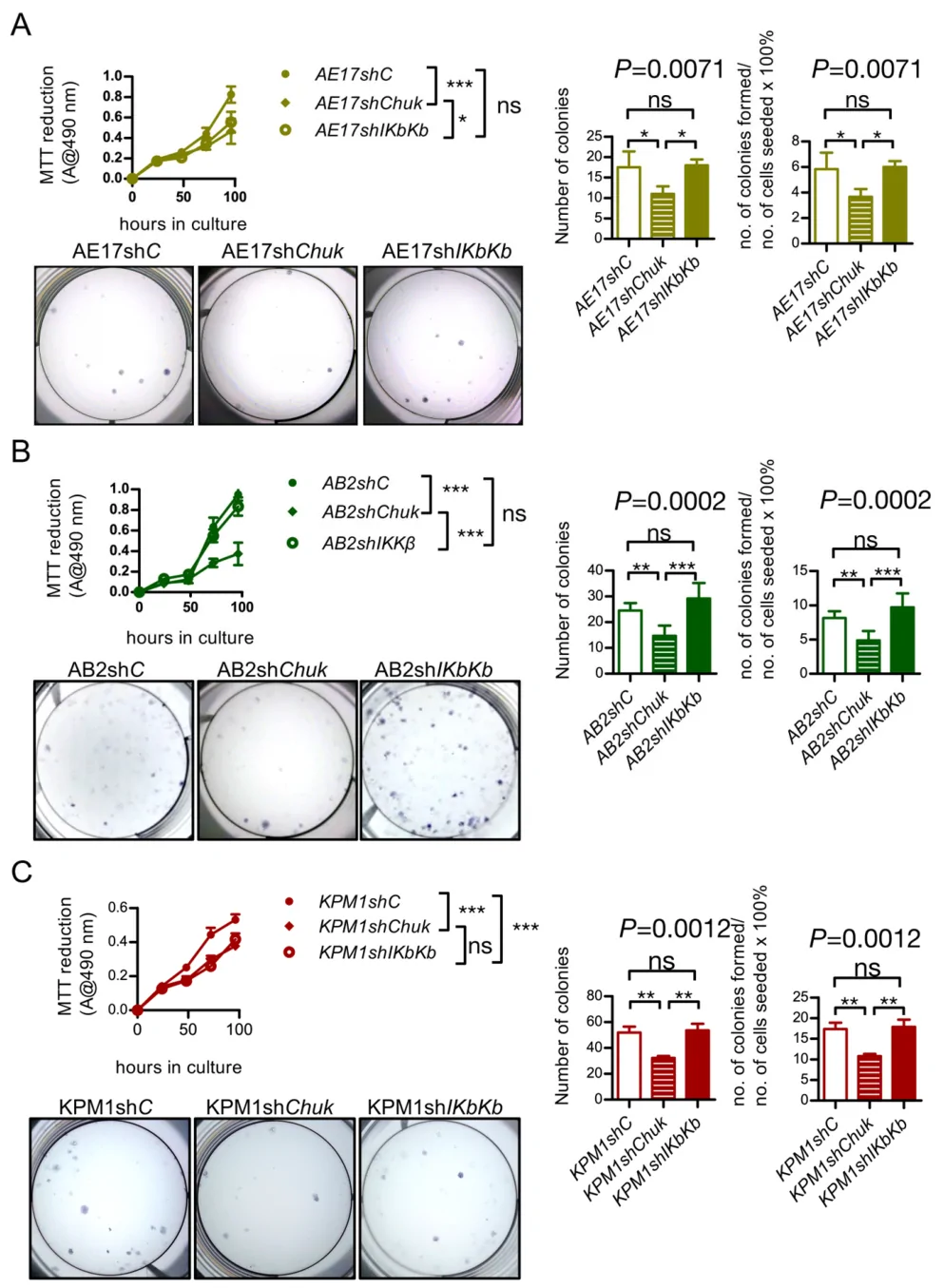
Effects of *Chuk* and *Ikbkb* silencing on tumor cell proliferation and clonogenicity. A metabolic growth assay (MTT) was used to identify changes in cell proliferation, and standard clonogenicity assays were used to determine colony formation in *Chuk*- and *Ikbkb*-silenced AE17 (**A**), AB2 (**B**) and KPM1 (**C**) cells. Data are expressed as mean ± 95% confidence intervals. Significances (*** *p* < 0.001, ** *p* < 0.01, * *p* < 0.05 and ns *p* > 0.05) were obtained following one- or two-way ANOVA with Bonferroni post hoc tests for MTT and colony formation assay, respectively.

We next investigated whether silencing IKKα and IKKβ gene expression had a differential impact on cell proliferation, as measured with a metabolic activity (MTT) assay, and on clonogenicity among our MPM cell models (Figure 4). Across all models, *Chuk* gene silencing was associated with reduced MTT signal relative to control, indicating reduced growth-associated metabolic activity. This measure has generally been well-correlated with a direct cell-count measure of proliferation in previous studies, but it is not a measure of cell number, per se. The reduction in MTT signal by *Chuk* silencing in AB2 cells, which are WT for both *Kras* and *Trp53,* (Figure 4B) and the lack of apoptosis (Figure 3) suggests that reduced metabolic growth and apoptosis may be mediated by different mechanisms. Silencing *Ikbkb*, on the other hand, significantly decreased the MTT signal only in the KPM1 cell model. Finally, colony formation was significantly decreased by *Chuk* signaling in all cell models, whereas *Ikbkb* silencing had no effect. These results suggest that, at least under our experimental conditions, *Chuk* signaling and, therefore, IKKα, has a significant impact on proliferation and colony formation in MPM cell models.

### 3.4. Effects of Chuk and Ikbkb Silencing on MPM Tumor Growth In Vivo

**Figure 5:**
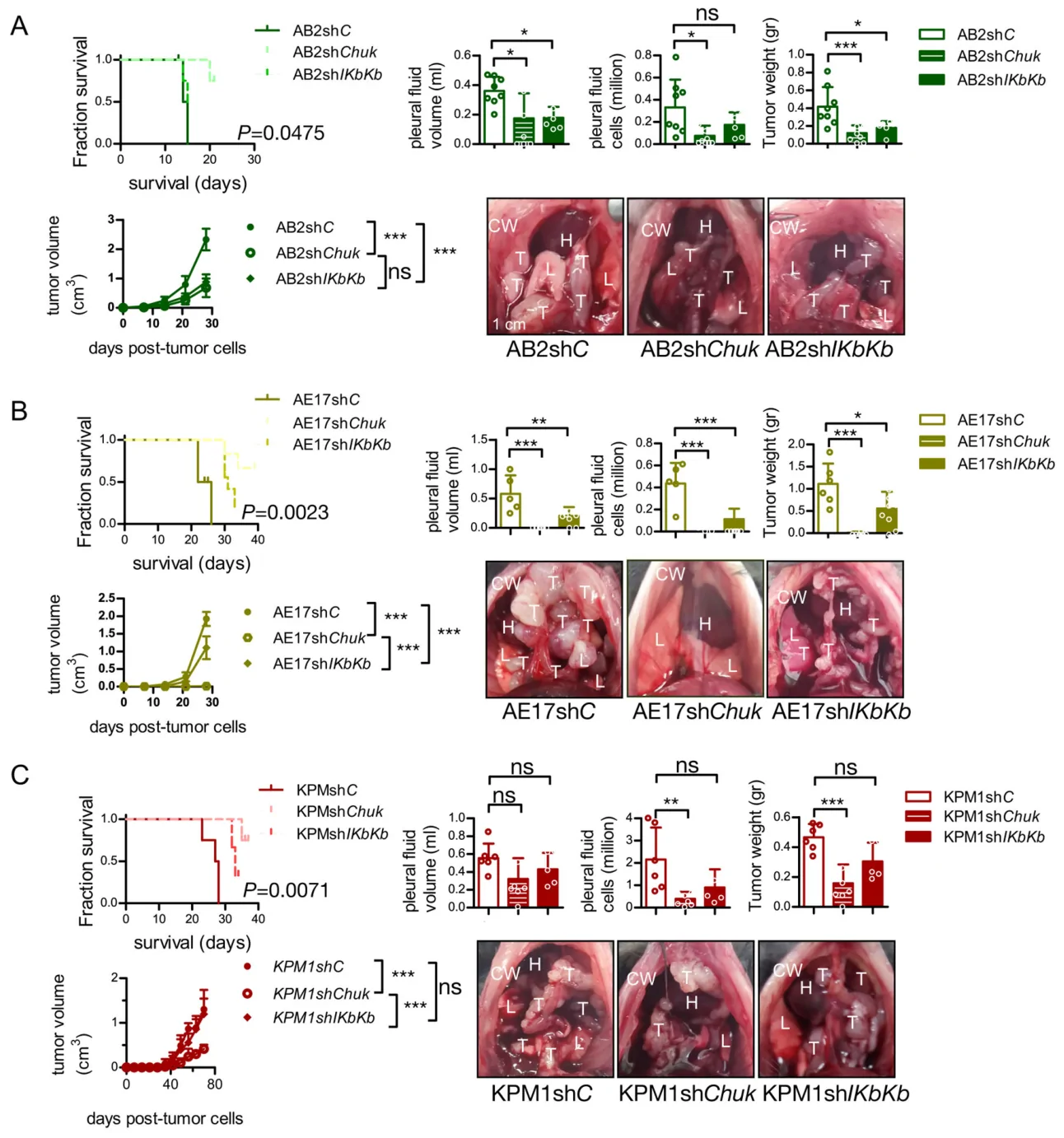
*Chuk* and *Ikbkb* silencing affects in vivo tumor development. *Chuk-* and *Ikbkb*-silenced MPM cell lines were delivered either intrapleurally or subcutaneously in BALB/c or C57BL/6 mice, as described in the method. The AB2, AE17 and KPM1 cell lines are shown in (**A**), (**B**), and (**C**), respectively. For each line, the survival curves are shown on the top left, and the tumor volumes of the subcutaneous tumors on the bottom left. The right-side panels show the results after intrapleural delivery of the MPM cells, with pleural malignancy parameters on the top right and representative pictures (T, tumors; L, lungs; CW, chest wall; H, heart) on the bottom right. The mouse group sizes are reported in Supplementary Table S4. Data are expressed as mean ± 95% confidence interval. Significances (*** *p* < 0.001, ** *p* < 0.01, * *p* < 0.05 and ns *p* > 0.05) were obtained after log-rank (Mantel–Cox) Tests for comparison of survival curves and one- or two-way ANOVA with Bonferroni post hoc tests for subcutaneous or intrapleural tumor parameters, respectively.

To further clarify the importance of IKKα and IKKβ in tumor growth associated with MPM, we next used the three shRNA-transduced murine cell models characterized above and monitored MPM progression following their instillation via either an intrapleural or a subcutaneous route. Survival was monitored and measurements of the development in tumor weight, pleural fluid accumulation and number of nucleated cells in pleural fluid were done as previously described [35]. Mice showed worse survival when injected with malignant cells in which IKKα and IKKβ expression were inhibited, with IKKα-inhibition being more effective. This is consistent with our in vitro findings showing that IKKα loss reduced cell proliferation ability and enhanced cancer cell death (Figure 5).

Following delivery of AB2 cells, which are WT for both *Kras* and *Trp53*, tumor size and pleural fluid formation decreased significantly regardless of whether the AB2 cells were transduced with *Chuk* or *Ikbkb* shRNA, whereas the number of nucleated cells in pleural fluid was significantly decreased only when *Chuk* was inhibited (Figure 5A). Similar results were seen in tumors derived from AE17 cells, characterized by *Kras*^G12C^ mutation (Figure 5B). Finally, KPM1-derived tumors showed significant decreases in growth parameters only when *Chuk*, but not *Ikbkb* was silenced, highlighting the involvement of IKKα signaling in tumors characterized by *Kras*^G12D^ mutation and *Trp53* loss of action (Figure 5C).

Finally, subcutaneous ectopic tumor-growth measurements confirmed the pleural tumor characteristics. Whereas both IKKα and IKKβ were important in tumors derived from AB2 and AE17 cells, only IKKα led to a statistically significant decrease in KPM1-derived tumors, further supporting the IKKα involvement in tumors characterized by *Kras*^G12D^ mutations and *Trp53* deletions (Figure 5C).

### 3.5. NF-κB-Signaling Gene Expression Is Associated with MPM in Human Tumors

**Figure 6:**
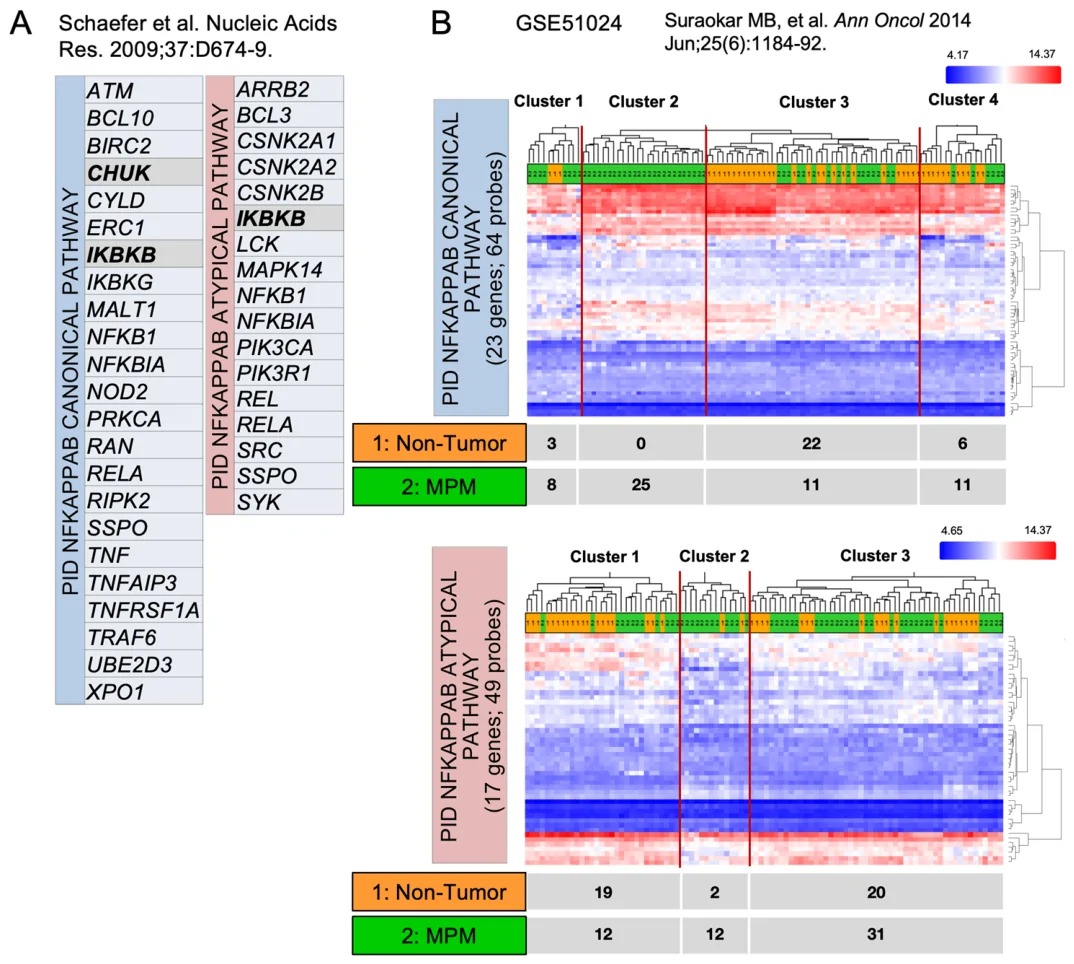
NF-κB gene expression pathways are represented in discrete clusters of human MPM. Two gene lists of 23 and 17 genes that represented the NF-κB canonical and atypical signaling pathways, respectively, ref. [48], were used as input for the analyses of human non-tumor and malignant pleural mesothelioma samples (**A**). Unsupervised hierarchical clustering of the gene expression in non-tumor and tumor samples revealed that the distribution of sample types in cluster 2 were significantly different than the other clusters when using either canonical or atypical gene sets as clustering input. The number of samples (non-tumor in green and tumor in orange) are listed under the heatmaps and were used in the subsequent analyses after the hierarchical clustering (**B**) [47]. Significances between clusters were obtained following χ2 and corrected Fisher-exact follow-up tests, *p*’s < 0.001, for canonical and atypical pathways respectively).

We next assessed whether NF-κB pathway-related genes were expressed in human MPM tumors. We chose two gene lists that were part of the Pathway Interaction Database [48] and that focused either on a combination of *Chuk* and *Ikbkb* (23 genes; canonical pathway) signaling or only on *Ikbkb* signaling alone (17 genes; referred to as atypical, but resembling the non-canonical pathway) (Figure 6A). Expression of these genes was identified in a publicly available data set (GEO Datasets accession ID: GSE51024) containing 55 MPM and 41 paired non-tumor lung parenchyma tissue samples from patients with MPM [47,48]. Unsupervised clustering, performed on the gene expression values of all samples, revealed that the canonical and atypical pathways resolved the samples into four and three clusters, respectively (Figure 6B). A follow-up analysis based on the number of non-tumor and tumor samples per cluster, showed that cluster 2 within both the canonical and the atypical pathways contained more tumor samples than the remaining clusters and characterized a subgroup of samples based on their gene expression. This suggests that, at least for some MPM patients, both NF-κB signaling pathways may be involved in MPM tumor formation. While this remains an exploratory association, similar analyses identifying other genes present in such sample clusters and whether they may correlate with disease progression of tumor mutation could provide further information about the mechanistic contribution of IKKα and IKKβ signaling in some MPM patients.

### 3.6. Transcriptional Responses Associated with Chuk- and Ikbkb-Silencing in Murine MPM Models

**Figure 7:**
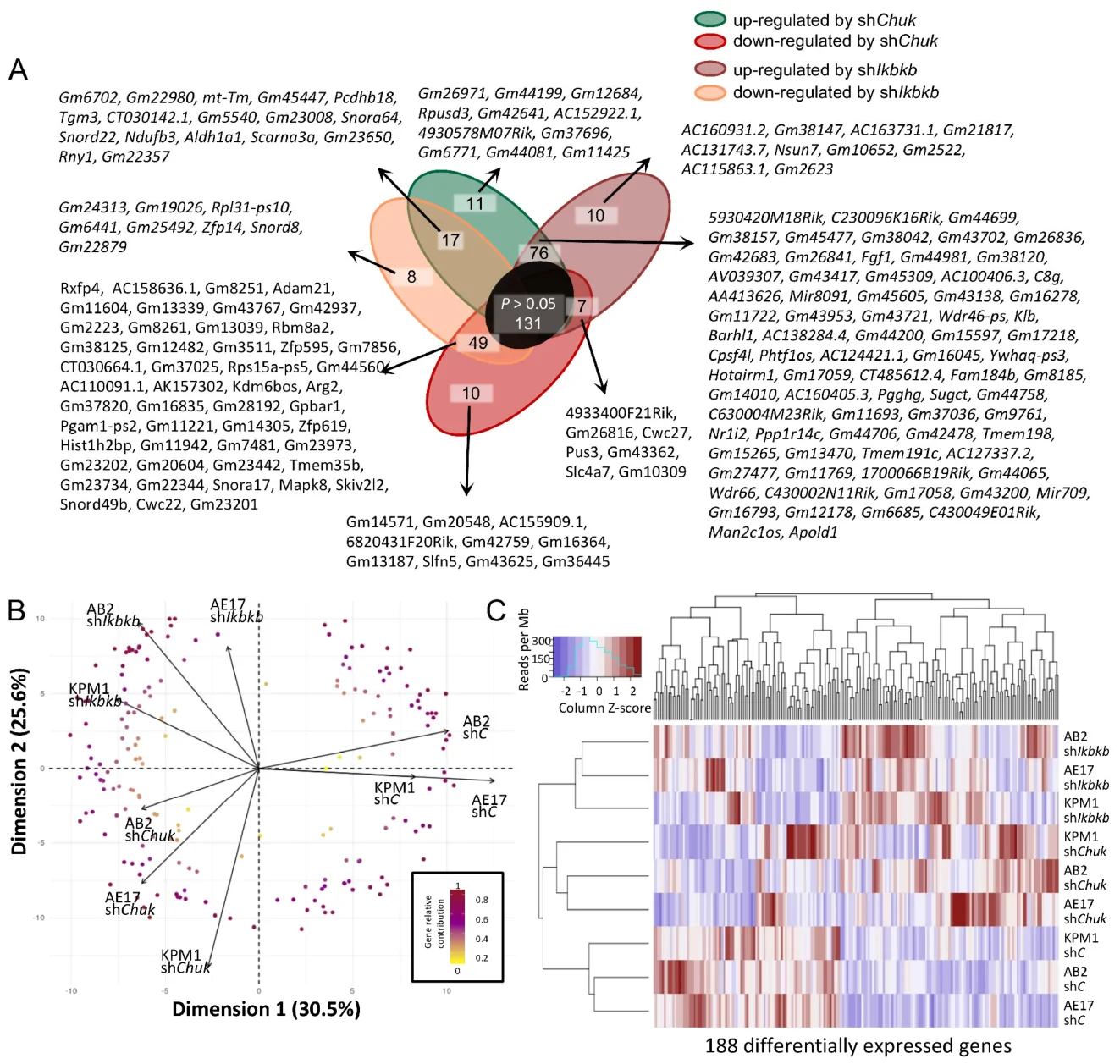
Exploratory transcriptional responses associated with *Chuk* and *Ikbkb* gene silencing in AB2, AE17 and KPM1 cell models. Venn diagram identifying differentially expressed genes, up- or down- regulated by *Chuk* or *Ikbkb* gene silencing (**A**). The central region labeled P > 0.05 in (**A**) contains 131 genes and was not interpreted as a significant response category. PCA biplot showing the relative gene contribution grouped by *Chuk* or *Ikbkb* silencing conditions and revealing the specificity of gene-silencing target (*Chuk*, *Ikbkb* or control) (**B**). Heatmap describing the unsupervised hierarchical clustering of the 188 differentially expressed genes by cell line and gene-silencing target (**C**).

RNA sequencing of *Chuk*- or *Ikbkb*-silenced AB2, AE17 and KPM1 cells revealed that 188 genes were differentially expressed (vs. control transfections; *p* < 0.05); 76 and 49 genes were upregulated or downregulated, respectively, when both *Chuk* and *Ikbkb* were silenced. Specific to each deletion, 28 and 66, and 86 and 74 genes were up- or down-regulated for *Chuk* or *Ikbkb*, respectively (Figure 7A). The clustering of the 188 DEGs specifically related to *Chuk*, *Ikbkb* of control transfections for each of the MPM cell lines (AB2, AE17 and KPM1) are shown as summaries in PCA biplots and heatmap (Figure 7B,C) and indicate that specific genes are associated with each experimental condition/cell line. These exploratory analyses identify specific sets of genes that may characterize the development of murine MPM and could be used as a starting point in identifying NF-kB-signaling-related therapeutic targets to be tested in future studies.

## 4. Discussion

This study is the first to investigate the involvement of IKKα and ΙΚΚβ kinases associated with NF-κB signaling in tumor formation using in vitro and in vivo MPM murine models. Based on the cytoplasmic vs. nuclear presence of the NF-κB-related signaling subunits (RelB and p52), there seems to be a predominance of the non-canonical activation pathway for both murine and human MPM cell models, but this finding needs to be further confirmed. We first used three mouse models of MPM, two chemically induced (AB2, AE17) and one genetically induced (KPM1), and showed that both IKKα and ΙΚΚβ were expressed. Their participation in disease progression was then explored by silencing *Chuk* and *Ikbkb,* the genes that encode IKKα and ΙΚΚβ, respectively. We found that both kinases were important for in vitro malignant cell proliferation. IKKα seemed to be highly associated with tumor cell survival in the presence of *Kras* mutations (AE17 and KPM1 cell models), suggesting a preferential involvement of the *Chuk* signaling pathway in the presence of this mutation. In vivo, both kinases were important for primary tumor formation. IKKα was significantly implicated in all MPM mouse models, whereas IKKβ impact in disease was more variable across models. Translating our results from a human cohort, we found that both canonical and atypical NF-κB signaling pathways that include *CHUK* and *IKBKB*, characterize a specific cluster of malignant samples, highlighting the importance of these genes as part of the mechanisms underlying MPM.

There is little evidence about the mechanistic implication of NF-κB in mesothelioma. One other study showed a possible protective role of both kinases in the survival and transformation of human mesothelial cells in malignant mesothelioma elicited by asbestos exposure-related TNF-α release [29]. Our findings support these previous observations and, because of we used of non-isogenic cell lines, raise the possibility that the contribution of IKKα to MPM cell survival may vary depending on the genetic background of the tumor cells. This is because AE17 and KPM1 cells carry different *Kras* alterations, with KPM1 cells also having a *Trp53* deletion and AB2 cells also being different in terms of strain background and tumor origin. Similarly, our exploratory transcriptomic analyses support the involvement of both canonical and atypical (non-canonical) NF-κB-associated gene signaling in MPM tumors, unfortunately without establishing specific diagnostic performance or tumor-cell-intrinsic *CHUK*/*IKBKB* activity. In-depth genomic, transcriptomic, multi-omic, and pharmacogenomic approaches may help refine future MPM classification and treatment prioritization, and prospective clinical validation remains necessary [11,12,13].

The activation of NF-κB signaling in exposure to asbestos and induced chronic inflammation is also associated with malignant transformations and resistance to chemotherapy [1,29,49]. So far, studies have focused on the inhibition of cell proliferation or the induction of apoptosis by targeting NF-κB-associated signaling pathways, such as in vivo SOCS1 gene delivery combined with cisplatin plus pemetrexed or in vitro ALDH1A3 expression modulation in the targeting of STAT3-NF-κB activity [49,50]. Moreover, the in vitro treatment of MPM cell lines with ranpirnase/Onconase, a cytotoxic antitumor ribonuclease, was reported to downregulate NF-κB/NFKB1-related signaling and decrease cell proliferation and invasion in MPM cell lines [51]. However, direct evidence regarding NF-κB in promoting mesothelioma formation has been insufficient. Only a few reports revealed NF-κB activation in mesothelioma cells in the presence of proteasome inhibitors or antitumor ribonuclease reagents (including IKKβ inhibitor IMD-0354) that may prevent NF-κB activity and lead to the effective inhibition of tumor proliferation and the induction of apoptosis [52,53,54,55]. However, these reagents may also influence the degradation and synthesis of a wide range of functional proteins. Therefore, the development of effective drugs with fewer side effects is necessary.

Although previous reports suggested an important role of NF-κB activation in mesothelioma [29,49,51,52], the evidence is sparse. Here, we show that both IKKα and ΙΚΚβ may be important in different aspects of cell proliferation and disease progression in vivo and possibly important in human malignancy as well. *Chuk*-signaling, more than *Ikbkb*-signaling, seemed associated with phenotypic changes in apoptosis, MTT reduction, clonogenicity, and several in vivo tumor development parameters. These findings suggest prioritizing further mechanistic investigation of the *Chuk*-linked phenotypes and the better characterization of the role of IKKα as a potential therapeutic target in MPM.

There are several limitations to this study. First, the gene-silencing experiments relied on single shRNA reagents that produced cross-target mRNA effects, without independent shRNAs or rescue experiments, thereby limiting target-specific attribution. Second, sh*Chuk*-associated apoptosis was observed in the two *Kras*-mutant cell lines but not in AB2, suggesting a potential relationship with *Kras* mutation and *Trp53* status that requires further validation in isogenic models. Third, knockdown was assessed primarily by qPCR and qualitative immunofluorescence, without quantitative immunoblotting or comprehensive functional analysis of NF-κB signaling, including IKK phosphorylation, IκBα degradation, p100 processing, NF-κB nuclear translocation, and transcriptional activity. These limitations prevent precise assignment of the tumor phenotypes as being dependent on IKKα or IKKβ signaling. Future studies should therefore incorporate independent shRNAs, genetic rescue, endpoint confirmation of target suppression, orthogonal readouts of canonical and non-canonical NF-κB signaling, validation of candidate transcriptional responses, and more appropriate human pleural controls.

## 5. Conclusions

In conclusion, the present study supports a role for IKK-associated signaling in the maintenance and progression of experimental MPM. Both IKKα and IKKβ contributed to cellular fitness and tumor development in selected settings, but IKKα depletion generated the most consistent effects on apoptosis, clonogenic survival and in vivo tumor growth. The findings support IKKα as a therapeutic candidate and raise the possibility of its interaction with oncogenic *KRAS* signaling.

## Data Availability

The publicly available human expression dataset analyzed in this study is available in the Gene Expression Omnibus under accession GSE51024. The RNA-sequencing data reported for the murine cell-line experiments are deposited under accession GSE129984.

## Supplementary Materials

The following supporting information can be downloaded at: https://www.mdpi.com/article/10.3390/cancers18182990/s1, Table S1: Lentiviral shRNA pools used for these studies; Table S2: Antibodies used for these studies; Table S3: PCR primers used for these studies; Table S4: Raw mouse group sizes for MPM mouse experiments; Figure S01: Figure 1C—RelA, cytosol (65 kDa); Figure S02: Figure 1C—RelA, nucleus (65 kDa); Figure S03: Figure 1C—RelB, cytosol and nucleus (68 kDa); Figure S04: Figure 1C—p52, cytosol (52 kDa); Figure S05: Figure 1C—p52, nucleus (52 kDa); Figure S06: Figure 1C—β-actin, cytosol (43 kDa); Figure S07: Figure 1C—β-actin, nucleus (43 kDa). Figure S08: Uncrpped image of Figure 1B.
