# Supplementary material for: Nuclear Factor-κB Signaling in Murine Models of Malignant Pleural Mesothelioma
Source: Cancers (Basel). 2026 Sep 16;18(18):2990. doi: 10.3390/cancers18182990 (PMC13605877; doi:10.3390/cancers18182990)
Supplement: Supplementary file 1 [file cancers-18-02990-s001.zip › cancers-4524504-S01-S07.pdf]

Whole Western Blot Images — Figure 1C

Manuscript ID: cancers-4524504

Whole visible blot/membrane regions are displayed in their archived orientation. Only outer scanner-bed/page margins were removed and proportional scaling was used; no tonal or content changes were made.

S01 Figure 1C—RelA, cytosol (65 kDa)

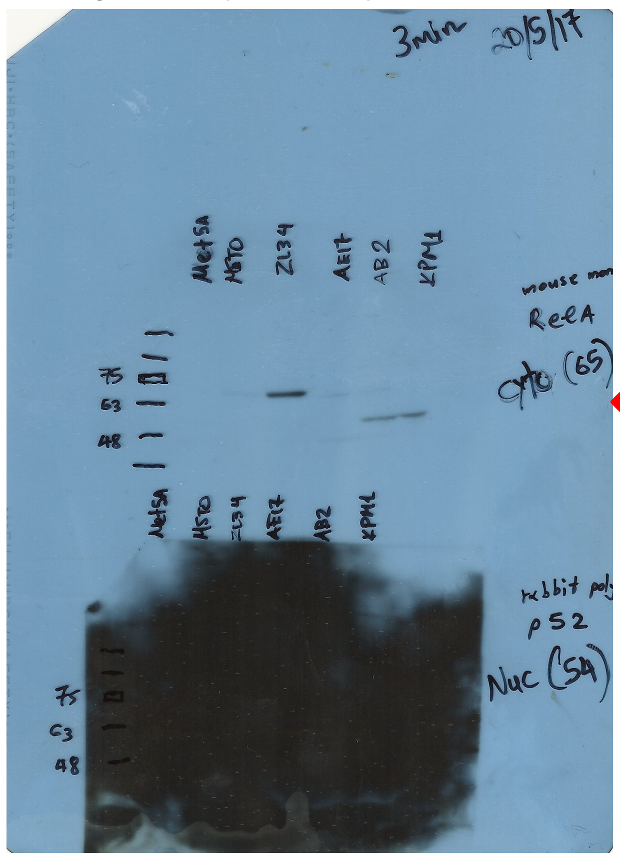

RelA, cytosol (65 kDa)

S02 Figure 1C—RelA, nucleus (65 kDa)

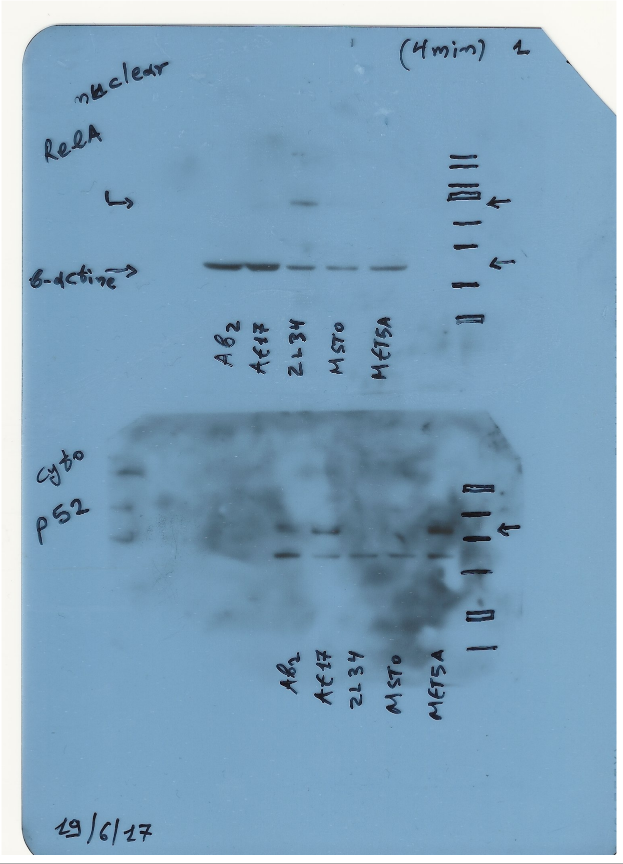

RelA, nucleus (65 kDa)

S04 Figure 1C—p52, cytosol (52 kDa)

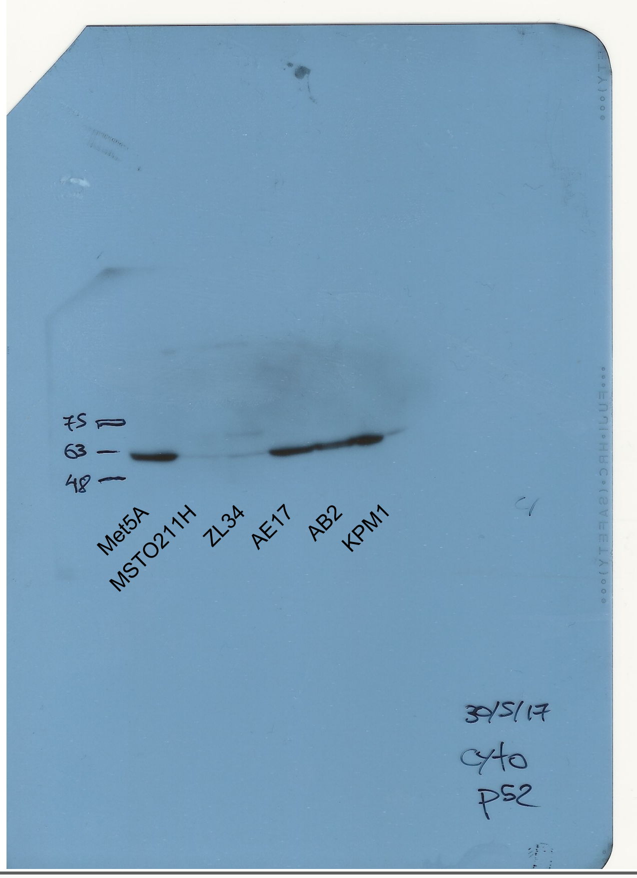

p52, cytosol (52 kDa)

S03 Figure 1C—RelB, cytosol and nucleus (68 kDa)

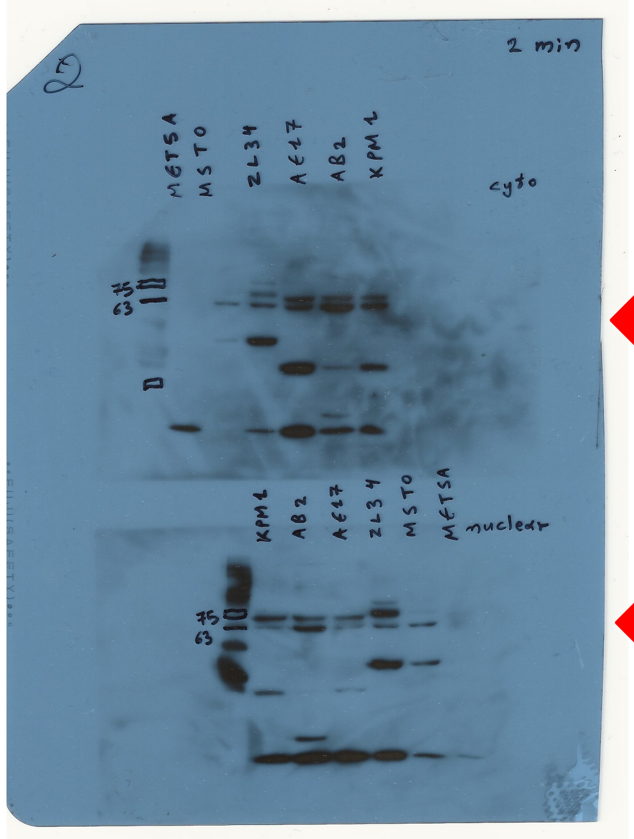

RelB, cytosol (68 kDa)

RelB, nucleus (68 kDa)

S05 Figure 1C—p52, nucleus (52 kDa)

Archived source: p52 nuclear.jpg

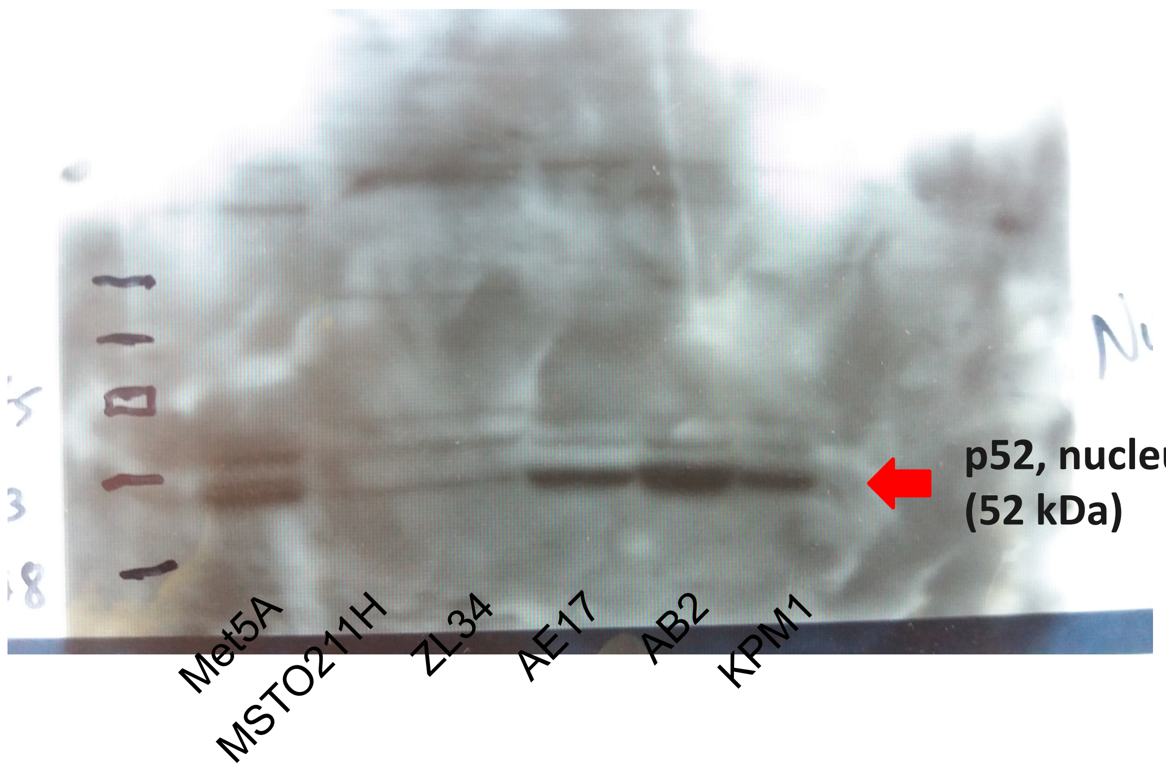

p52, nucleus (52 kDa)

S06 Figure 1C—β-actin, cytosol (43 kDa)

Archived source: IKKB and actin for mesothelial cell lines.JPG

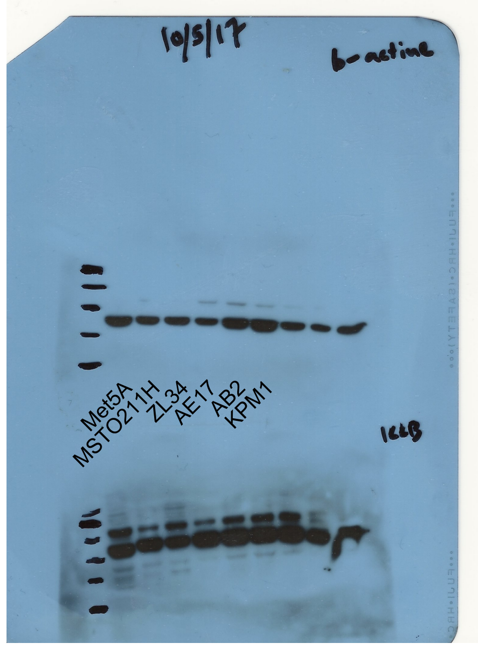

β-actin, cytosol (43 kDa)

S07 Figure 1C—β-actin, nucleus (43 kDa)

Archived source: b-actin.tif

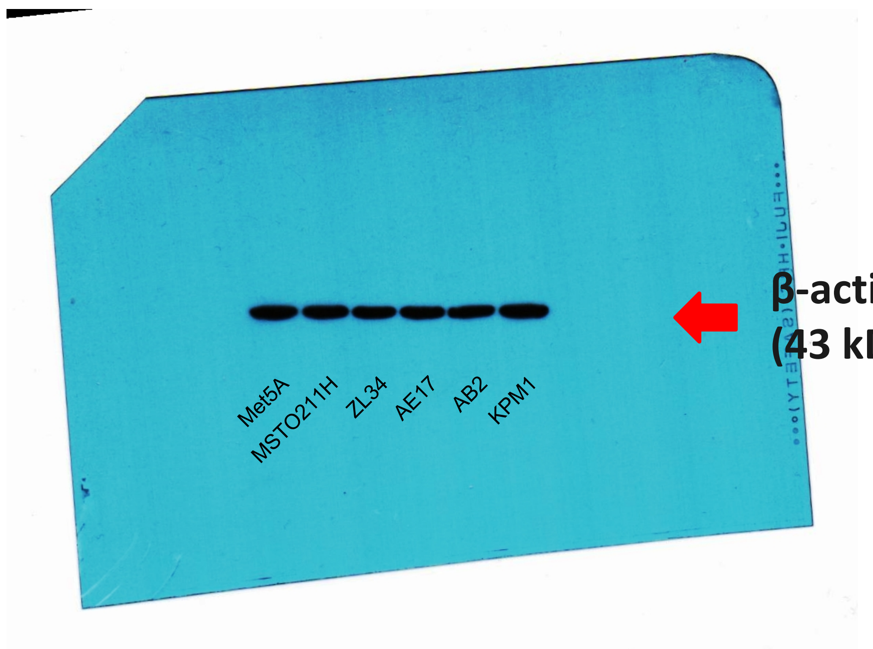

β-actin, nucleus (43 kDa)
