# Supplementary material for: Nuclear Factor-κB Signaling in Murine Models of Malignant Pleural Mesothelioma
Source: Cancers (Basel). 2026 Sep 16;18(18):2990. doi: 10.3390/cancers18182990 (PMC13605877; doi:10.3390/cancers18182990)
Supplement: Supplementary file 1 [file cancers-18-02990-s001.zip › cancers-4524504-supplementary.pdf]

Supplementary Table S1. Lentiviral shRNA pools used for these studies

Target	Abbreviation	Catalog #	Target sequences
random	sh*C*	sc-108080-V	target sequence proprietary
Chuk	sh*Chuk*	sc-29366-V	-CCATGGTGTTTGAATGTATTT<br>-CTCTCAGTGTGTTCTAGATTT<br>-GCAAGCAGAAGATTATTGATT
Ikbkb	sh*Ikbkb*	sc-35645-V	-GATGACATCTTGAACTTGATT<br>-CTGCACATTTGAATCTGTATT<br>-CAGCTCTCTTAGACAGTTATT

Provider: Santa Cruz Biotechnology, Dallas, TX, USA.

Supplementary Table S2. Antibodies used for these studies

Method[a]	Target	Provider[b]	Catalog #	Dilution	Conjugate[c]
WIB	RelA	Santa Cruz Biotechnology	sc-8008	1/200	-
WIB	RelB	Santa Cruz Biotechnology	sc-226	1/250	-
WIB	p52	Santa Cruz Biotechnology	sc-298	1/250	-
WIB	β-actin	Santa Cruz Biotechnology	sc-47778	1/500	-

IHC, IF	IKKα	Santa Cruz Biotechnology	Sc-7606	1/100	-
IHC, IF	IKKβ	Invitrogen	10AG2	1/100	-
WIB	Goat anti-rabbit IgG	Southern Biotech	4030-05	1/80000	HRP
WIB	Goat anti-mouse IgG	Santa Cruz Biotechnology	sc-516102	1/2000	HRP
IF	Donkey anti-rabbit and anti-mouse IgG	Invitrogen	A21206 and A21202	1/1000	Alexa 488

[a]Application: IF, immunofluorescence; WIB, Western immunoblotting

[b]Providers: Santa Cruz Biotechnology, Dallas, TX, USA; Southern Biotech, Birmingham, AL, USA; Invitrogen, Carlsbad, CA, USA. [c]Conjugated visualization target for secondary antibodies: HRP, horse radish peroxidase.

Supplementary Table S3. PCR primers used for these studies.

Method[a]	Primer	Sequence	Amplicon (bp)
RT-PCR	Trp53F	GTAGCTTCAGTTCATTGGGA	1450
RT-PCR	Trp53R	GAAGTCATAAGACAGCAAGGA	
qPCR	ChukF	AACCAGCCTCTCAGTGTGTT	106
qPCR	ChukR	CTGGATGCAAATGGTCCTTCA	
qPCR	IkbkbF	CAGTGCCTGTGACAGCTTAC	115
qPCR	IkbkbR	TTGCTCCTTCACAGTGTCCT	
qPCR	GusbF	TTACTTTAAGACGCTGATCACC	165
qPCR	GusbR	ACCTCCAAATGCCCATAGTC	
PCR	Mycoplasma Spp.F	GGGAGCAAACAGGATTAGATACCCT	270
PCR	Mycoplasma Spp.R	TGCACCATCTGTCACTCTGTTAACCTC	

[a]Application: PCR, DNA polymerase chain reaction; qPCR, quantitative (real-time) PCR.

Provider: VBC Biotech, Vienna, Austria

Supplementary Table S4. Raw mouse group sizes for MPM mouse experiments.

**Experiment**	**Cell line**	**Recipient strain**	**Raw number[a], shC / sh*Chuk* / sh*Ikbkb***	**Total number**
Intrapleural MPM	AB2	BALB/c	8 / 10 / 7	25
Intrapleural MPM	AE17	C57BL/6	6 / 6 / 9	21
Intrapleural MPM	KPM1	C57BL/6	6 / 8 / 5	19
Subcutaneous tumor	AB2	BALB/c	5 / 5 / 4	14
Subcutaneous tumor	AE17	C57BL/6	5 / 4 / 5	14
Subcutaneous tumor	KPM1	C57BL/6	6 / 6 / 5	17

Abbreviations and notes: MPM, malignant pleural mesothelioma; shC, control shRNA; sh*Chuk*, *Chuk*/IKKα-targeting shRNA; sh*Ikbkb*, *Ikbkb*/IKKβ-targeting shRNA. [a]Raw number= number of unique individual mouse records and is reported in the order shC / sh*Chuk* / sh*Ikbkb.*
